# Keeping Control: The Role of Senescence and Development in Plant Pathogenesis and Defense

**DOI:** 10.3390/plants4030449

**Published:** 2015-07-13

**Authors:** Eva Häffner, Sandra Konietzki, Elke Diederichsen

**Affiliations:** 1 Freie Universität Berlin, Fachbereich Biologie, Chemie, Pharmazie, Institut für Biologie, Dahlem Centre of Plant Sciences, Angewandte Genetik, Albrecht-Thaer-Weg 6, 14195 Berlin, Germany; 2 Norddeutsche Pflanzenzucht H.G. Lembke KG, Hohenlieth, D-24363 Holtsee, Germany; E-Mails: loni.cera@web.de (S.K.); elked@zedat.fu-berlin.de (E.D.)

**Keywords:** senescence, development, pathogen, resistance, biotroph, necrotroph, hemibiotroph, *Verticillium*, signaling, gene expression

## Abstract

Many plant pathogens show interactions with host development. Pathogens may modify plant development according to their nutritional demands. Conversely, plant development influences pathogen growth. Biotrophic pathogens often delay senescence to keep host cells alive, and resistance is achieved by senescence-like processes in the host. Necrotrophic pathogens promote senescence in the host, and preventing early senescence is a resistance strategy of plants. For hemibiotrophic pathogens both patterns may apply. Most signaling pathways are involved in both developmental and defense reactions. Increasing knowledge about the molecular components allows to distinguish signaling branches, cross-talk and regulatory nodes that may influence the outcome of an infection. In this review, recent reports on major molecular players and their role in senescence and in pathogen response are reviewed. Examples of pathosystems with strong developmental implications illustrate the molecular basis of selected control strategies. A study of gene expression in the interaction between the hemibiotrophic vascular pathogen *Verticillium longisporum* and its cruciferous hosts shows processes that are fine-tuned to counteract early senescence and to achieve resistance. The complexity of the processes involved reflects the complex genetic control of quantitative disease resistance, and understanding the relationship between disease, development and resistance will support resistance breeding.

## 1. Introduction

**Figure 1:**
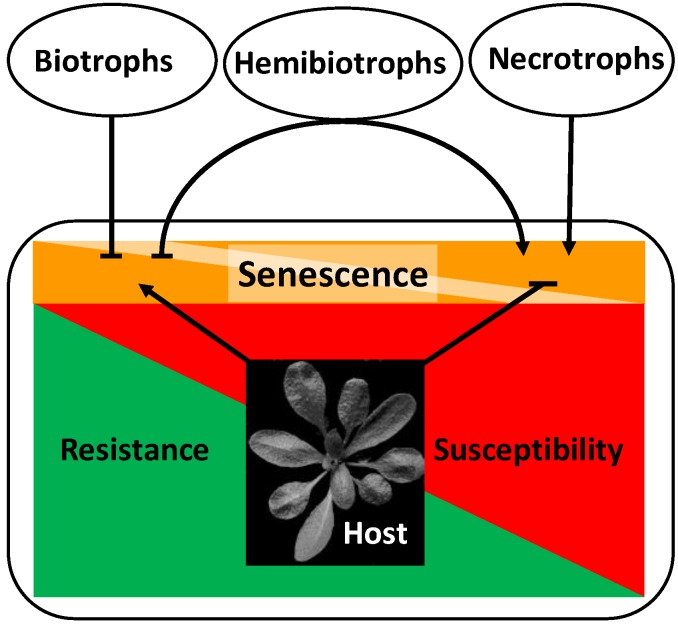
Relationship between senescence and resistance/susceptibility in necrotrophic and biotrophic host-pathogen interactions. Biotrophic pathogens and hemibiotrophs in their biotrophic stage inhibit senescence to increase susceptibility. The host can control pathogen growth and promote resistance by activating senescence-like processes. Necrotrophic pathogens and hemibiotrophs in their necrotrophic stage induce senescence to increase susceptibility. The host can control pathogen growth and promote resistance by inhibiting senescence-like processes. Hemibiotrophs switch from a biotrophic to a necrotrophic lifestyle in the course of their development. Arrows stand for activation/induction, bars for inhibition.

Plant senescence, the developmental program leading to nutrient remobilization and finally the degradation and death of tissue, can be triggered by many factors, both intrinsic and extrinsic [1]. Pathogen infections interact with developmental processes in a complex way. The outcome of this interaction may greatly influence plant productivity as well as resistance and susceptibility. Pathogenesis and development mutually influence each other. On the one hand, developmental conditions of the host plant may determine the outcome of pathogen infection. On the other hand, pathogen infection can change the developmental program of the host [2]. Symptoms of senescence often accompany the progression of disease. In other cases, senescence is delayed in response to pathogen infection. The lifestyle of the pathogen plays an important role for the developmental response of the host [3,4]. Necrotrophic pathogens derive nutrients from dead cells, whereas biotrophic pathogens need living cells to meet their nutritional demands. Between both extremes, a third nutritional type is recognized: the hemibiotrophs. Hemibiotrophs adopt both lifestyles, switching from a biotrophic phase at the beginning of the infection to a necrotrophic lifestyle as pathogenesis proceeds [5]. Generally, senescence and cell death are regarded as favorable to the necrotrophic lifestyle, and necrotrophic pathogens have adopted strategies to induce senescence by manipulating the respective signaling pathways in plants [4]. Consequently, a defense strategy against necrotrophs would consist of the host’s ability to prevent senescence-like processes. Conversely, biotrophic pathogens benefit from living cells and change signaling processes in the host to delay senescence. The most efficient defense strategy against biotrophs is therefore the controlled induction of cell death during the hypersensitive response (HR), thus starving the pathogen in confined regions. It can easily be imagined that the situation is especially complex for hemibiotrophs. Resistance against necrotrophs and hemibiotrophs is often quantitative, *i.e.*, restricting, but not eliminating the pathogen. Figure 1 gives an overview on developmental modifications of hosts by pathogens with different lifestyles and the respective resistance reactions of the host.

The constantly growing knowledge about the molecular mechanisms controlling development and pathogen response provides us with the opportunity to recognize patterns and strategies that promote resistance and increase productivity of the host plant. Intriguingly, the same signaling molecules often control both senescence and pathogen defense. Especially in quantitative disease resistance, molecular responses to pathogen infection are often difficult to interpret: They may be induced by the pathogen for its own benefit, or they may be defense reactions of the host. Studies using resistant and susceptible genotypes as well as mutants and transgenes contribute to disentangling the complex network of defense and development. It becomes more and more obvious that the respective signaling pathways that are triggered by relatively few and simple molecules have multiple functions, that they are cross-linked in multiple ways and that individual branches can be switched on or off by both host and pathogen. Major interest lies on components that represent regulatory nodes and points of convergence, since they may be decisive for resistance or susceptibility.

In the present article, the interrelation between pathogenesis and senescence is treated on two levels: In Chapter 2, current knowledge about major players in the control of senescence and pathogen defense is reviewed. Signaling pathways and their role in senescence and defense against pathogens are described and regulatory nodes and pathway integrators are highlighted. Chapter 3 gives examples of host-pathogen-interactions with strong developmental implications—to see “the players in action” and learn about control strategies of plants and pathogens. Focus is laid on pathogenesis caused by necrotrophic and hemibiotrophic pathogens and its interaction with plant senescence. Complex mutual influence of pathogenesis and development is observed in the interaction between the hemibiotrophic vascular pathogen *Verticillium longisporum* and its cruciferous hosts. The most obvious developmental implication, premature senescence, is at the same time the most problematic disease symptom caused by *V. longisporum* in oilseed rape crops, leading to massive yield losses [6]. Over the past years, we have collected data giving a precise description of the interaction between development and pathogenesis/resistance in *Brassica* spp. and in *Arabidopsis thaliana*. We have used *A. thaliana* to address the underlying molecular mechanisms. Some of our published and unpublished data on the development-pathogenesis-defense-interplay are included in the present review.

## 2. Major Players in the Control of Senescence and Pathogen Response

Among the most important players in the control of senescence and pathogen defense are phytohormones like ethylene (ET), jasmonic acid (JA), salicylic acid (SA) and abscisic acid (ABA), and to a minor extent brassinosteroids (BR) as hormones that promote senescence. Cytokinins, auxins, and gibberellins can be regarded as counteracting hormones that delay senescence. Transcription factors play a pivotal role in all signaling networks as they target the responsive genes. They often act as regulatory nodes between signaling branches and thus contribute to the fine-tuning of developmental or defense reactions. Reactive oxygen species (ROS) control senescence and cell death as well as the early events after pathogen recognition. Since the signaling networks behind these key players are each highly complex and cross-linked among each other, mainly those modules relevant for senescence and pathogen response and nodes of interaction and integration will be pointed out. If not mentioned otherwise, all reviewed studies used the model plant *Arabidopsis thaliana*.

### 2.1. Phytohormones

#### 2.1.1. Ethylene

The gaseous plant hormone ET controls many developmental processes as well as responses to environmental stimuli [7]. Its role in the interaction with necrotrophic and hemibiotrophic pathogens is characterized by two opposite effects: ET primarily promotes senescence [8,9] and is involved in programmed cell death during HR [10]. Cell ageing and death is beneficial to necrotrophic pathogens. Accordingly, inducing or synthesizing ET is a virulence strategy applied by many necrotrophic or hemibiotrophic pathogens (e.g., [11,12,13]). However, ET interacts synergistically with JA in the activation of defense against necrotrophic pathogens, thereby promoting resistance [14].

The rate-limiting step of ET biosynthesis is catalyzed by 1-AMINOPROPANE-1-CARBOXYLATE (ACC-) synthases (ACS). In *A. thaliana*, nine ACS isoforms exist that are differentially regulated to adjust ET levels to changing needs [15,16]. ET is perceived in *A. thaliana* via five different receptors located in the endoplasmic reticulum [17,18]. In the absence of ET, they activate the serine-threonine-kinase CONSTITUTIVE TRIPLE RESPONSE 1 (CTR1), which in turn represses the activity of ETHYLENE-INSENSITIVE 2 (EIN2) by phosphorylation [19]. Upon the binding of ET to the receptors, CTR1 is inactivated and EIN2 mediates the stabilization of the transcription factors EIN3 and EIN3-LIKE 1 (EIL1) in the nucleus [20,21]. EIN3 and EIL1 activate a great diversity of primary responsive genes like ET response factors (*Erf*). ERF transcription factors induce secondary responsive genes, thus modulating a great variety of developmental and stress-induced responses [22].

The senescence-promoting effect of ET has long been known [23,24]. ET is neither required nor sufficient to induce senescence, but it can accelerate the process depending on different genetic and environmental conditions. Some of the genes modulating the plant’s receptiveness to ET-mediated induction of senescence have been identified [9,25]. A mechanism by which ET accelerates leaf senescence has been elucidated only recently [26]: The central transcription factor EIN3 has been shown to repress the transcription of micro-RNA miR164, thereby allowing the accumulation of its target gene product, the ARABIDOPSIS NAC DOMAIN CONTAINING PROTEIN 92 (ANAC092)/ORESARA 1 (ORE1), a transcription factor that positively regulates leaf senescence [27,28].

The activation of defense responses against necrotrophic pathogens, which is characterized by the production of PLANT DEFENSIN 1.2 (PDF1.2), requires JA and ET. Both hormones together induce transcriptional activation of the ET response factors ERF1 and OCTADECANOID-RESPONSIVE ARABIDOPSIS AP2/ERF 59 (ORA59) via EIN3 and EIL1 [29,30]. Generally, ET contributes to resistance against necrotrophic pathogens, and mutation of central signaling components results in enhanced susceptibility [10], but there are exceptions (e.g., [31]). In some cases, disrupting different components of the ET signaling pathway had opposite effects on the outcome of the infection. Even different receptor mutants can show very different pathogen responses: While *ein4* mutants of *A. thaliana* showed increased susceptibility to the hemibiotrophic fungus *Verticillium longisporum* [32], *etr1* mutants were more resistant than wild type [32,33]. Initially, the ET receptors were regarded as functionally redundant [34]; however, recent evidence supports the hypothesis that different receptor combinations can produce different outputs depending on the tissue and the ET concentration [35].

#### 2.1.2. Jasmonic Acid

Much like ET, JA plays a prominent role in the control of senescence, cell death and in resistance to necrotrophic pathogens. JA is generated by lipid peroxidation in chloroplasts and peroxisomes. The physiologically active compound, the amino-acid conjugate jasmonoyl-isoleucine (JA-Ile) acts by de-repressing its target genes [36]. The JA-Ile receptor is a complex consisting of a JASMONATE ZIM DOMAIN (JAZ)-protein and the F-box-protein CORONATINE INSENSITIVE 1 (COI1). In the absence of JA-Ile, JAZ-proteins act as repressors of target genes by binding transcription factors such as MYC2 or EIN3. Upon recruitment by COI1, JAZ proteins are ubiquitinated by the SKP1-CULLIN-F-BOX- (SCF)-complex and targeted for degradation by the 26S proteasome. Transcription factors are released and target genes are transcribed [37,38,39].

The induction of leaf senescence was the first effect on plant development that was described for JA [40]. Exogenous application of methyl jasmonate can induce senescence in *A. thaliana* leaves and JA-levels rise during senescence. The facts that several JA biosynthesis genes are up-regulated during senescence and that the *coi1*-mutant was not responding to JA-induced senescence confirmed a regulatory role of the hormone in senescence [41]. Recent research has revealed some of the underlying molecular mechanisms. JA levels have been shown to be developmentally controlled by the transcription factors TCP2, 4 and 10. They exerted a direct effect on JA biosynthesis via transcriptional control of LIPOXYGENASE 2 (LOX2) [42]. JA signaling acts synergistically with ET as EIN3 and EIL1 are interactors of JAZ [30]. Release of JAZ repression of EIN3 and EIL1 allows downstream processes to occur (see above). The final proof whether this JA/ET synergism during defense reactions also contributes to the induction of senescence is still missing [43]. Another key factor in the JA-mediated induction of senescence has been shown to be RUBISCO ACTIVASE (RCA), which is transcriptionally down-regulated in a COI1-dependent manner [44]. Typical markers for senescence, like chlorophyll degradation and the expression of senescence-associated genes (SAGs) were associated with RCA-down-regulation. *rca*-mutants showed an early senescence phenotype and reduced transcription of JA-regulated genes [44]. Furthermore, JA has been shown to promote programmed cell death by inducing the production of ROS in mitochondria [45]. In another context, JA acted as a negative regulator of senescence: JA has been shown to induce the expression of the ETIOSPECIFYING SENESCENCE REGULATOR/ETIOSPECIFYER PROTEIN (ESR/ESP). This protein had an inhibitory effect on the senescence-promoting transcription factor WRKY53, which in turn was induced by SA. In the absence of WRKY53, ESR/ESP mediated the conversion of glucosinolates to nitriles, leading to enhanced resistance against *Alternaria brassicicola and Pseudomonas syringae* [46].

Synergism between the defense activation pathways controlled by JA and ET is mainly characterized by the induction of *Pdf1.2* via ERF1 and ORA59 [29]. This pathway is antagonized by two other major defense responses: One of them is triggered upon insect attack and wounding and requires synergism between JA and ABA. In this branch of JA signaling, transcription factor MYC2 is activated, which leads to expression of defense genes against insect attack as represented by the marker gene *Vegetative storage protein 2* (*Vsp2*). Antagonism takes place at the phytohormone level (ABA *vs.* ET) and at the transcription factor level (MYC2 *vs.* ERF1 and ORA59) [47]. The second signaling cascade repressing the ERF/ORA59-branch of JA-signaling is typically triggered by biotrophic pathogens and is based on the induction of SA upon recognition of pathogen-associated molecular patterns (PAMPs) or pathogen effectors (see below). Multiple molecular components mediate the antagonistic crosstalk between JA- and SA- activated defense pathways to achieve a high degree of flexibility in the fine-tuning of adequate defense reactions [47]. Besides this antagonism, JA has an important role in the establishment of systemic acquired resistance (SAR) [48].

#### 2.1.3. Salicylic Acid

SA is primarily known as a defense hormone [49], but it has multiple roles in development as well [50]. SA has been shown to promote developmental senescence via *npr1* and *phytoalexin-deficient 4* (*pad4*) [51]. Furthermore, it has been shown to induce the transcription factor WRKY53, an important positive regulator of senescence and pathogen defense [52]. The signaling pathway leading to PATHOGENESIS-RELATED (PR) gene induction and SAR during PAMP-triggered immunity (PTI) and effector-triggered immunity (ETI) is the backbone of SA-mediated defense responses [49]. Activation of the transcriptional co-activator NONEXPRESSOR OF PR-GENES (NPR1) leads to the expression of a large set of defense-related genes such as PR1 and several WRKY transcription factors, and to the establishment of SAR [47]. Independent of NPR1, SA is also required for HR [53], during which controlled localized cell death occurs with participation of ROS [49]. During HR, biotrophic pathogens are encapsulated at the infection site and prevented from spreading to healthy parts of the plant.

#### 2.1.4. Abscisic Acid

ABA has perhaps the most multi-faceted role in senescence regulation and in the control of defense against pathogens. Whereas ABA is generally regarded as a senescence-promoting hormone, its role in pathogen defense is ambiguous for biotrophic as well as necrotrophic pathogens [54]. Crosstalk with many other signaling pathways and cellular processes such as JA-, ET-, SA-, cytokinin- and sugar signaling, ROS production and signaling as well as autophagy exists [47,55,56,57,58].

ABA is perceived by receptor complexes consisting of an RCAR (REGULATORY COMPONENTS OF ABA RECEPTORS)/PYR1 (PYRABACTIN RESISTANT 1)/PYL (PYR-LIKE) protein [59,60] and a PROTEIN PHOSPHATASE 2C (PP2C), like ABA INSENSITIVE 1 (ABI1) and ABI2. In *A. thaliana*, 14 RCAR isoforms and nine RCAR-interacting PP2C-isoforms have been identified that can interact with each other in a combinatorial way, possibly addressing different downstream signaling components [61,62]. Downstream signaling involving SNF1-RELATED KINASEs (SnRKs) like OPEN STOMATA 1 (OST1) leads to the targeting of ion channels in the guard cells and to transcriptional activation or repression of target genes via transcription factors like ABI5 [62,63].

ABA plays a crucial role in developmental as well as stress-induced senescence [64]. During developmental senescence, ABA levels rise (e.g., [65,66]), and ABA biosynthesis genes are up-regulated [67]. ABA-inducible RECEPTOR PROTEIN KINASE 1 (RPK1) has been shown to play an important role in developmental leaf senescence [68]. ABA furthermore mediates the connection between senescence and carbohydrate metabolism: By down-regulating extracellular invertase via INHIBITOR OF CELL WALL INVERTASE (INVINH), ABA reduces sink strength of tissues. INVINH induction proved to be essential for ABA-induced senescence in tomato [69]. Cytokinins counteract ABA in the modulation of sink-source-relationships by inducing extracellular invertase and thus delaying senescence [70]. Extracellular invertase plays a crucial role in phloem unloading and providing sink tissues with carbohydrates [71]. ABA-responsive transcription factors that control senescence have also been identified: ATAF1 was shown to integrate H_2_O_2_- and ABA-induced senescence and acted upstream of ANAC092 [72]. Interestingly, the NAC transcription factor VND-INTERACTING 2 (VNI2) was activated by ABA under salinity stress and negatively regulated senescence. As a bifunctional transcription factor, it has been proposed to act as an integration point between senescence and stress regulatory pathways [73]. Cross-talk of ABA and SA in inducing developmental senescence is suggested by experiments with the *A. thaliana* mutant *saul1* that showed early developmental senescence in low light. The U-BOX ARMADILLO E3 UBIQUITIN LIGASE 1 (SAUL1) presumably targets the ABA-degrading enzyme AAO3 for degradation by the 26S-proteasome [74]. SA and PAD4- but not NPR1-dependent signaling were required for the early-senescence phenotype of *saul1* [75]. The PAD4-pathway is also required for cell death during HR.

ABA can have positive and negative effects on defense responses, which depends mainly on the pathogen. Mostly, ABA negatively affects resistance [76,77,78,79,80]. Accordingly, the hormone is produced by many pathogens, both biotrophic and necrotrophic, as a virulence factor [54]. ABA suppresses defense against biotrophs by antagonizing SA-production and the NPR1-branch of SA-signaling [81,82]. Potentially, also the JA/ET branch of JA-signaling can be antagonized by promoting the antagonistic JA-ABA branch [47]. Resistance-promoting effects of ABA were mainly observed in interactions with necrotrophic pathogens [83]. A positive effect of ABA on defense responses was exerted by priming for callose deposition [84]. Furthermore, ABA has been shown to play an important role in H_2_O_2_-homeostasis by controlling CATALASE 1- (CAT1) induction and H_2_O_2_-production [85]. As described below, ROS like H_2_O_2_ play an important role in early defense responses under controlled conditions, but become highly toxic at higher concentrations [3].

#### 2.1.5. Brassinosteroids

BR are important plant growth hormones and have a senescence-promoting effect [86]; plants defective in BR biosynthesis like the *A. thaliana* mutant *de-etiolated 2* (*det2*) or the BR receptor mutant *brassinosteroid insensitive 1* (*bri1*) showed delayed senescence [87,88]. BR induced resistance against the (hemi-)biotrophic pathogens tobacco mosaic virus (TMV), *Oidium* and *Pseudomonas syringae* in tobacco and *Xanthomonas oryzae* and *Magnaporthe grisea* in rice [89]. BR have been shown to induce PR1 in an NPR1-independent way [90]. Furthermore, potato plants sprayed with BR became more resistant to *Phytophthora infestans* and showed increased ABA- and ET levels [91]. The important BR signaling components BRI1-ASSOCIATED RECEPTOR KINASE 1 (BAK1) and BAK-LIKE 1 (BKK1) play a role in cell death regulation, however, in a way that was independent of BRI1 and BR [87].

#### 2.1.6. Cytokinins, Auxins, Gibberellins

Whereas all the aforementioned phytohormones generally have senescence-promoting effects, cytokinins, auxins and gibberellins are regarded as more or less senescence-retarding hormones [55]. While few studies addressed the role of gibberellins in senescence (e.g., [92,93]), the evidence for **cytokinin**-induced delay of senescence is paramount [55,86,94]. Tobacco plants transformed with the *Isopentenyl*-*phosphotransferase* (*Ipt*-) gene, which encodes the rate-limiting enzyme for cytokinin biosynthesis, under the promoter of *Senescence-associated-gene 12* (*Sag12*) displayed a functional stay-green phenotype with prolonged photosynthetic activity [95]. Functional stay-green genotypes with an enhanced cytokinin status were also more resistant to drought [94,96]. Surprisingly, transgenic tobacco plants over-expressing *Cytokinin oxidase* (*Ckx*)-genes with a lower endogenous cytokinin content did not show early senescence [97,98]. It was concluded that cytokinin does not control the onset of senescence but prevents its execution [86].

**Auxins**, mainly represented by indole acetic acid (IAA), control many aspects of plant growth and development [99]. Auxins are synthesized in young tissues from tryptophan via two different pathways, highlighting their exceptional importance [100]. Another distinct feature is the polar auxin transport by auxin efflux carriers with polar distribution in cells. It is a prerequisite for the fine auxin gradients shaping plant architecture [99]. Auxin signaling shows many parallels to JA signaling: Upon auxin binding, the F-box protein TIR forms a complex with AUX/IAA repressors, targeting them for ubiquitination and subsequent degradation [101,102]. AUXIN RESPONSE FACTORs (ARF) are released and initiate the transcription of target genes [99]. Auxins hold a complex role in senescence. While in some studies a senescence-retarding effect of auxins was found, others suggest a role in its induction [86]. Exogenously applied auxin was shown to delay senescence [103], and over-expression of the auxin biosynthesis gene YUCCA6 caused elevated auxin levels and extreme longevity [104]. Furthermore, *arf2* mutants that are defective in the auxin signaling repressor ARF2 showed delayed senescence [105]. Antagonistic cross-talk of auxin- and JA-signaling has been discovered by Jiang *et al.*, with WRKY57 acting as a regulatory node which was competitively up-regulated by auxin and down-regulated by JA. WRKY57 delayed senescence [106]. While these findings support a senescence-retarding role of auxin, it is unexpected that auxin biosynthesis genes were up-regulated and contents of free auxin rose during senescence [107,108]. In addition, auxin has been found to act synergistically with ET in the induction of senescence via SENESCENCE-ASSOCIATED RECEPTOR-LIKE KINASE (SARK) in soybean and in *A. thaliana* [109]. Likewise, over-expression of the auxin responsive *Small auxin-up RNA gene 36* (*Saur36*) led to an early-senescence phenotype of *A. thaliana*, suggesting a positive regulation of senescence by auxin [110].

The role of the growth-promoting hormones in pathogen defense has long remained cryptic. During the last decade many surprising roles of gibberellins, cytokinins and auxins in biotic stress response have been described, often in the context of synergism or antagonism with known stress signaling pathways [47,111,112]. Gibberellins target DELLA repressors for degradation [113]—a signaling pathway that is analogous to the one for JA and auxin signaling. Several DELLA proteins compete with MYC2 in binding to JAZ repressors [114], thereby facilitating JA-signaling. GA-mediated DELLA degradation thus leads to repression of JA-signaling. Recently, GbWRKY1 transcription factor has been shown to dampen JA-response in the course of *Verticillium dahliae* infection in cotton, thereby de-repressing GA-signaling [115]. The mutual repression of JA- and GA-signaling reflects the antagonism between growth and defense that controls resource allocation [116,117]. Through the JA-SA-antagonism, gibberellin signaling acts synergistically with SA, promoting resistance to biotrophs [118]. Likewise, cytokinins have been shown to act synergistically with SA via ARABIDOPSIS RESPONSE REGULATOR 2 (ARR2), which is recruited by SA-controlled transcription factors to enhance expression of PR1 [111].

Auxin has been shown to interact antagonistically with SA, thereby increasing susceptibility to biotrophic pathogens [112]. SA has been found to repress the auxin receptor TIR1, thus stabilizing the AUX/IAA-repressors of auxin signaling [119]. This is consistent with the finding that PR1 is hyperinducible by SA in auxin receptor mutants [120]. Furthermore, SA has been shown to induce WES1/GH3.5, an enzyme that conjugates IAA to amino acids, thus reducing free IAA levels [121]. *A. thaliana* plants over-expressing WES1 were more resistant to *P. syringae* and showed elevated PR1 levels [121]. A similar observation was made in rice, where reduction of free IAA by over-expression of the conjugating enzyme OsGH3.8 resulted in enhanced resistance against *Xanthomonas oryzae* pv. *oryzae*, which was, however, SA-independent. It was hypothesized that increased resistance was due to inhibition of expansins [122]. Strikingly, a different study found a direct disease-promoting effect of an IAA-aspartate conjugate (IAA-Asp) via induction of microbial virulence factors [123]: Exogenously applied IAA-Asp promoted disease symptoms caused by *Botrytis cinerea* and *P. syringae* DC3000, and the enzyme GH3.2, which conjugates IAA to aspartate, was up-regulated by infection with these pathogens. Accordingly, *gh3.2* mutants were more resistant against both pathogens. Other IAA-conjugating GH3-enzymes showed minor or no effects on pathogenesis in this study. While the disease-promoting role of auxin in interactions with biotrophic pathogens is relatively clear, its role in hemibiotrophic and necrotrophic interactions is ambiguous. It was shown that auxin signaling is required for quantitative resistance against the necrotrophs *B. cinerea* and *Plectosphaerella cucumerina*, as *auxin resistant* mutants *axr1*, *axr2* and *axr6* were more susceptible to these pathogens [124]. Interestingly, *axr6* also has an important role in JA-signaling, since it encodes CULLIN1, a component of the SCF complex interacting with COI1 [125]. However, *axr1* and *axr2* showed increased resistance to *Fusarium oxysporum*, suggesting a disease-promoting role of auxin signaling for this pathogen [126]. In the same study, auxin transport was shown to mediate susceptibiliy.

### 2.2. Transcription Factors

The vital importance of transcription factors in regulating developmental processes and stress responses has already been emphasized in the context of the signaling pathways described above. The most relevant groups in the control of senescence and defense comprise WRKY transcription factors [127], APETALA 2/ETHYLENE RESPONSIVE ELEMENT BINDING PROTEINS (AP2/EREBP) [128], NAM-ATAF1,2-CUC2 (NAC) [129,130], R2R3-MYB [131], basic HELIX LOOP HELIX proteins (bHLH) [132] and C2H2-type zinc finger transcription factors [133]. In this section, the focus will be on selected WRKY transcription factors, because they are often not attributed to individual phytohormone signaling pathways. Instead, many of them have an integrative function for diverse incoming signals. Hence, some WRKY transcription factors play a pivotal role as nodes of convergence in the regulation of both senescence and defense against pathogens [52,134]. WRKY transcription factors constitute a gene family comprising 75 members in *A. thaliana* with diverse functions in development and responses to biotic and abiotic stress [127,135].

WRKY transcription factors with a confirmed role in senescence include WRKY6, WRKY53, WRKY70 and WRKY30 [136,137]. WRKY6 expression increased during senescence and it strongly induced SENESCENCE-INDUCED RECEPTOR-LIKE KINASE/FLG22-INDUCED RECEPTOR-LIKE KINASE (SIRK/FRK1), a gene induced during senescence and also during PAMP-triggered immunity [137]. WRKY53 was up-regulated during senescence and underlay a negative feedback loop. WRKY53 over-expression accelerated senescence, and knockout lines showed delayed senescence [138]. WRKY30 was expressed during developmental leaf senescence and both WRY53 and WRKY30 were shown to be responsive to ROS [136]. WRKY70 is a negative regulator of senescence. Despite their contrasting role in regulating senescence, WRKY53 and WKY70 both were shown to be positive regulators of defense with a role in SAR [134,136]. WRKY38 and 62, on the other hand, acted as negative regulators of SA- and NPR1- mediated basal defense [139]. Another regulatory node is formed by WRKY18, 40 and 60. They were shown to form homo- and heterocomplexes with each other to establish a highly plastic system to respond to different microbial pathogens [140]. Triple knockout mutants showed enhanced resistance to biotrophic *P. syringae*, but increased susceptibility to necrotrophic *B. cinerea*. The mutant phenotype correlated with increased *PR1*-transcript levels and decreased *Pdf1.2* transcript levels, emphasizing the role of these three transcription factors in mediating JA/ET-induced defenses. All three factors are also negative regulators of ABA-signaling. Especially WRKY40 repressed the transcription factor *Abi5* [141]. Conversely, WRKY8 is described as a positive regulator of ABA- and a negative regulator of ET signaling [142].

### 2.3. Reactive Oxygen Species, Programmed Cell Death and Autophagy

ROS like hydrogen peroxide, the superoxide anion or the hydroxyl ion function as signaling molecules during senescence and pathogen response, but also as antimicrobial compounds and as toxins leading to programmed cell death and necrosis [143,144,145]. Programmed cell death and autophagy, key processes in senescence and defense against pathogens, are both responsive to ROS [144,145]. It was suggested that three levels of ROS activity can be distinguished in plant cells depending on the concentration: At low levels, ROS can be controlled by the antioxidant machinery of the plant. At medium levels, ROS change the redox status of the cell in a way that leads to programmed cell death, while at high, toxic concentrations, cells are killed in an uncontrolled way, leading to necrosis [145]. ROS originate from different sources in the plant: They are a by-product of both photosynthesis and respiration and their concentration is usually controlled by antioxidant enzymes such as catalases, superoxide dismutases, peroxidases and glutathione-S-transferases, and antioxidant compounds like ascorbic acid and glutathione [146,147]. As signaling molecules, ROS are produced in a controlled way by the RESPIRATORY BURST OXIDASE HOMOLOGUE (RBOH)-NADPH-oxidases [148,149,150].

ROS signaling is closely linked to developmental events. During senescence, the antioxidant capacity of cells decreases and ROS concentrations increase [151,152]. Conversely, ROS have been shown to induce leaf senescence [153,154]. Important senescence-promoting transcription factors like ANAC092/ORE1 [155], ATAF1 [72], ORE1-SISTER 1 (ORS1) [156] or WRKY53 [138] were found to be up-regulated by H_2_O_2_. Recently, the NAC transcription factor ATAF1 has been identified as an integration point cross-linking H_2_O_2_- and ABA-signaling in the control of leaf senescence [72].

Rapid biphasic ROS production follows pathogen recognition during PTI and ETI and is termed oxidative burst [157]. Defense reactions triggered by ROS include direct effects like oxidative reinforcement of cell walls by cell-wall peroxidases [158]. More importantly, multiple signaling processes leading for example to the production of antimicrobial compounds are triggered by ROS [159]. Interestingly, ANAC042/JUB1, a NAC transcription factor which is induced by H_2_O_2_, had a negative regulatory effect on senescence [154] and has been shown to be involved in camalexin induction [159]. It may have a role in the fine-tuning between senescence- and defense signaling by H_2_O_2_. Ca^2+^ is not only required to trigger ROS production by RBOHs [160,161], but is also involved in downstream signaling processes [162]. MITOGEN-ACTIVATED PROTEIN KINASE-(MAPK-) signaling cascades are activated by ROS [161,163], such as MPK6, which is a major integrator of senescence and defense regulatory pathways [36,49,55,164]. SA, JA and ET are induced by ROS [143] and the respective signaling pathways are tightly cross-linked. A prominent example is the redox sensitivity of NPR1, which is reduced after a compensatory rise of glutathione levels following the oxidative burst. Reduction leads to monomerization of NPR1, thus facilitating SA-signaling [165]. ROS furthermore triggered the ABA-mediated closure of guard cells [162], and ABA has been shown to induce H_2_O_2_ via MAPK-signaling [58]. While the aforementioned hormone signaling pathways are mainly reinforced by ROS, an inhibitory effect of ROS on auxin signaling has been reported, leading to the so-called “stress-induced morphogenetic response” [166]. ROS are highly mobile signals and are thus an essential component of systemic responses like SAR [148,163].

HR involving programmed cell death is one of the most efficient defense reactions initiated by ROS [149,167]. However, the benefit of HR for the host strongly depends on the lifestyle of the pathogen [3,168]: HR is a particularly efficient strategy against biotrophic pathogens, as it leads to confined lesions at the sites of pathogen entry. Being dependent on living cells, the pathogen is prevented from spreading. A strategy of biotrophic pathogens therefore consists in the induction of antioxidants, leading to ROS scavenging and the inhibition of HR [169]. HR can also be efficient against necrotrophs when it is associated with the deposition of antimicrobial metabolites such as callose or lignin at the entry sites of the pathogen. However, being a pro-death strategy, cell death and HR can also facilitate the growth of necrotrophic pathogens, and ROS induction leading to cell death in the host can be a virulence strategy [170]. In this case, the capacity of the host to produce antioxidants would promote resistance. Programmed cell death is closely linked to autophagy, a process by which cell constituents are enclosed in vesicles (autophagosomes) that fuse with the vacuole. Recent studies support the view that autophagy, though induced upon senescence, is a pro-survival strategy of cells which is necessary for controlled nutrient recycling during senescence and stress [171]. *A. thaliana atg* mutants, which are deficient in autophagy, show premature senescence, are hypersensitive to oxidative stress and hypersusceptible to necrotrophic pathogens [172]. The hypersusceptibility to *B. cinerea* infection could be explained by an interaction of ATG18a with WRKY33, which mediates defense against necrotrophic pathogens [173]. There are indications that autophagy negatively regulates HR-PCD [174]. Accelerated cell death in *atg* mutants depends on SA signaling via NPR1 [175]. In other studies, a cell death-promoting effect of autophagy has been found [176]. Interestingly, the role of autophagy in HR-PCD depended on the type of R gene-signaling during ETI: While HR induction via TIR-NBS-LRR receptors depended on autophagic processes, HR mediated by CC-NBS-LRR was largely independent of autophagy [176].

Taken together, the evidence suggests a positive role of ROS in defense against necrotrophic pathogens as long as their production is tightly controlled by redox buffering and signaling processes that prevent cell death and necrosis.

## 3. Developmental Implications of Host-Pathogen-Interactions

### 3.1. Host Development Affects Pathogenesis

The following examples demonstrate that developmental conditions or developmental stages of the host plant can affect the outcome of the host-pathogen interaction. Examples of developmentally conditioned susceptibility and resistance are presented for pathogens with different lifestyles.

#### 3.1.1. Senescence-Like Processes Confer Quantitative Resistance against Biotrophic Pathogens in Cereals

Three examples from cereals demonstrate that senescence-like processes can confer resistance against devastating biotrophic pathogens.

*Lr34*, a gene belonging to the pleiotropic drug resistance family of ABC-transporters, is a resistance gene from wheat that confers broad-spectrum-, durable and quantitative resistance to the biotrophic pathogens *Puccinia triticina*, *P. striiformis* and *Blumeria graminis* [177]. The resistance conferred by *Lr34* has strong developmental implications: It works only in adult plants during the grain-filling phase and it is connected with senescence-like processes. Resistance was highly correlated with a morphological trait called leaf-tip necrosis, which was most pronounced in the uppermost leaf, the flag leaf [177]. Genes up-regulated in the presence of the *Lr34*-gene comprised ABA-inducible genes, genes associated with cold and drought stress and with seed maturation. Typical defense genes were not differentially expressed [178]. It was suggested that senescence-like processes are the cause for increased resistance [177].

A second gene conferring quantitative resistance against *P. striiformis* in wheat is *Yr36*/WHEAT KINASE START 1 (WKS1) which is present in some wild wheat species, but not in hexaploid bread wheat [179]. WKS1 contains a lipid-binding START domain and a kinase domain. The protein exists in six splice variants, only one of which is full-length, while the others have truncated START domains. The full-length transcript is up-regulated by *P. striiformis*-infection, while the other transcripts are not [179]. Full-length WKS1 was shown to phosphorylate THYLAKOID-ASSOCIATED ASCORBATE PEROXIDASE (tAPX), thus reducing its capacity to detoxify H_2_O_2_ [180]. Transgenic wheat containing multiple copies of *Wks1* showed accelerated senescence even without infection. It was concluded that WKS1 facilitates cell death that is slower than HR cell death, thus allowing restricted pathogen growth [180].

In the third example, *Mlo* from barley, coding for a membrane protein with seven transmembrane domains [181] delayed senescence and conferred susceptibility to biotrophic *B. graminis* f. sp. *hordei* (Bgh), but increased resistance against the hemibiotroph *Magnaporthe grisea* and the necrotroph *Bipolaris sorokiniana*. *mlo* mutants showed accelerated leaf senescence, were more susceptible against *M. grisea*, but highly resistant against Bgh. This resistance was associated with rapid necrosis formation and H_2_O_2_- production at the infection sites [182]. *Mlo* is an inducible gene that is up-regulated by a number of pathogen and stress treatments [182] and controls basal resistance [183].

#### 3.1.2. Late-Senescing Genotypes Are More Resistant to Necrotrophic and Hemibiotrophic Pathogens

A late-senescing genotype may confer quantitative resistance to necrotrophic and some hemibiotrophic pathogens. However, these effects are mostly multigenic and individual genes are hard to identify. In cowpea (*Vigna unguiculata*), resistance against the necrotrophic pathogen *Macrophomina phaseolina* was associated with late maturity, and two *M. phaseolina* resistance QTL co-localized with QTL mediating late maturation [184]. It was shown that *M. phaseolina* infection in the legume model plant *Medicago truncatula* induced genes that lead to inactivation of auxin and suppression of auxin signaling [185]. Exogenous application of auxin could alleviate disease symptoms. To date it is not known whether the identified resistance QTL [184] are involved in auxin homeostasis or signaling.

Crucial events of pathogenesis often depend on developmental stages of the host. For example, mature-green tomatoes are more resistant to infection by the necrotrophic pathogen *B.cinerea* than red ripe fruits [186]. Gene expression analysis showed how developmental and pathogenesis effects were intricately interwoven for each of the involved hormone signaling networks controlled by ET, SA, ABA and JA [186]. Generally, ET seemed to have a disease-promoting effect in tomatoes by accelerating maturity. Down-regulation of the central signaling components LeEIL3 and LeEIL4 is interpreted as a plant strategy to dampen the disease-promoting effect of ET. LeERF1, which is a crucial component of the PDF1.2-branch of JA/ET-signaling, was up-regulated during infection. In contrast, the regulation of ABA synthesis and signaling components pointed to a clear disease-promoting role of this hormone in the process of ripening. More resistant, mature green tomatoes showed up-regulation of the ABA-response inhibitor RECEPTOR FOR ACTIVATED C KINASE 1 (RACK1) [186]. 

**Figure 2:**
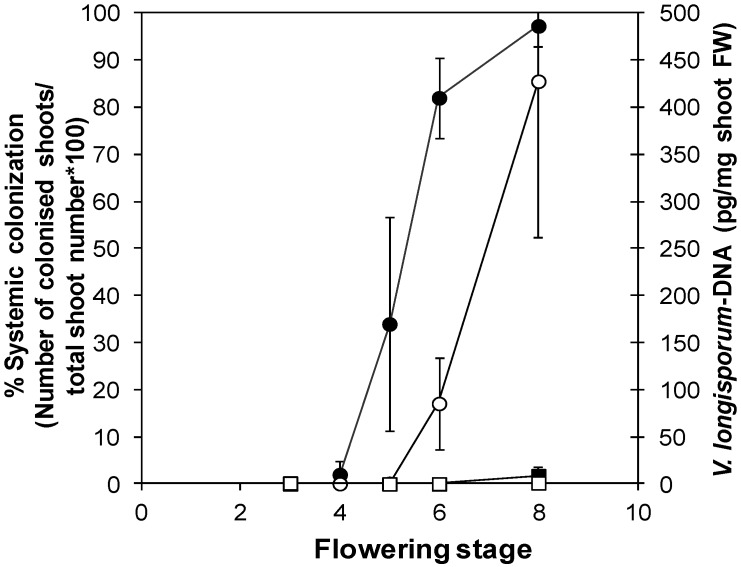
Systemic colonization of L*er* and Bur by *V. longisporum* in the course of plant development. Percentage of colonized apical shoot segments in a plating assay (closed symbols) and fungal DNA-content determined by qPCR in apical shoot segments (open symbols) in L*er* (circles) and Bur (squares) at different developmental stages. Developmental stages: 3 = 1–3 flowers open, 4 = 4–10 flowers open, 5 = more than 10 flowers open, 6 = up to 3 siliques mature, 7 = 4–6 siliques mature, 8 = up to 50% of siliques on main shoot mature. *N* = 4 (Bur) or 5 (L*er*), one replicate corresponded to a batch of 30 plants. Error bars denote standard deviations (data from [188]).

In the interaction between *V. longisporum* and its cruciferous hosts, the onset of flowering is a key event affecting pathogenesis. In susceptible *Brassica* genotypes, the phase of systemic colonization through the xylem was triggered by the onset of flowering [187]. The time-course of *V. longisporum*-colonization in the susceptible *A. thaliana* ecotype Landsberg *erecta* (L*er*) showed the same pattern (Figure 2, [188]). Systemic colonization of the shoot system, which can be monitored by re-isolating the pathogen from apical shoot segments plated on malt agar, started with the onset of flowering. Extensive fungal proliferation, which was detected by quantifying fungal DNA-contents via qPCR, started at the onset of silique maturity. Figure 2 also shows that the resistant *A. thaliana* ecotype Burren (Bur) was barely colonized in the course of its development. 

**Figure 3:**
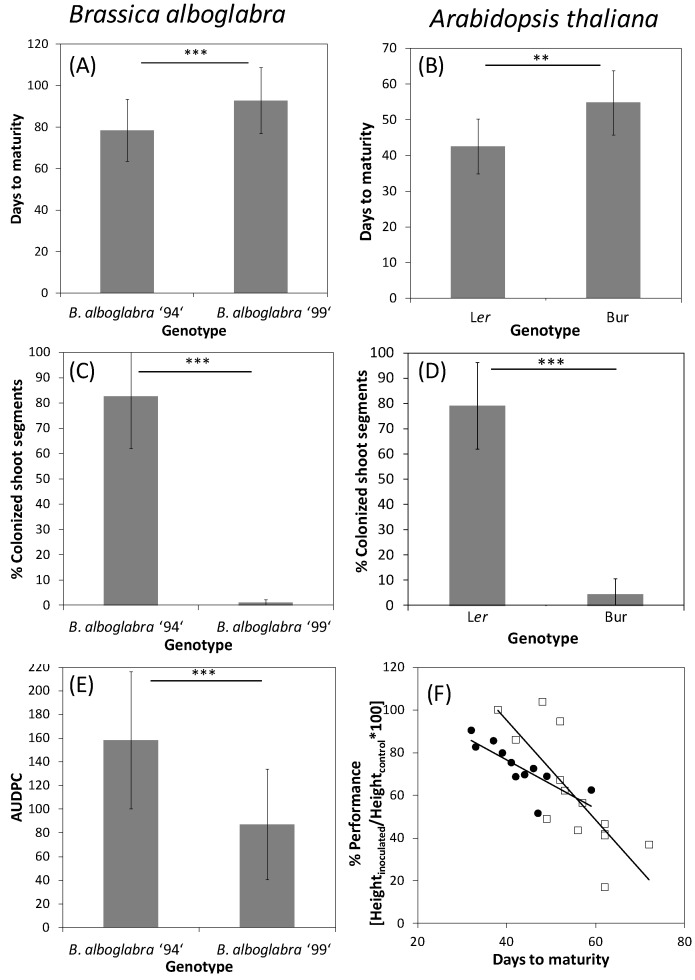
Developmental type and *V. longisporum* disease parameters of two accessions of *B. alboglabra* (left side) and *A. thaliana* (right side). (**A**,**B**) Development time of mock-inoculated control plants from inoculation to maturity (*B. alboglabra*) or germination to maturity (*A. thaliana*). (**A**) Single-plant data pooled from three independent experiments, *N* = 58 (94), *N* = 59 (99); (**B**) Average values from means of 9 and 11 independent experiments comprising at least 15 plants each; (**C**,**D**) Systemic colonization by *V. longisporum*. (**C**) *N* = 5; (**D**) *N* = 9 (L*er*), *N* = 11 (Bur); (**E**) Area under the disease progress curve (AUDPC, [191]) of *V. longisporum*-inoculated plants. High values stand for fast disease progression. Single-plant values from six independent experiments, *N* = 243 (94), *N* = 253 (99); (**F**) Performance of L*er* (circles) and Bur (squares) after *V. longisporum* infection depending on development time (data from [188]). Development was prolonged in winter experiments under lower light intensities. Performance (%) = Height_inoculated_/Height_control_ *100. Each data point represents a separate experiment comprising at least 30 plants. Experiments were performed at different seasons over three years under greenhouse conditions. Correlations: L*er*: *r* = −0.79, *p* = 0.004; Bur: *r* = −0.79, *p* < 0.001. Error bars denote standard deviations, asterisks denote significant differences below the 0.01 (**) and 0.001 (***) significance levels respectively (Student’s *t*-test).

Resistance against *V. longisporum* in *A. thaliana* as well as in *Brassica* was also often associated with a late-flowering developmental type (Figure 3A,B). In *B. alboglabra*, the late-flowering accession “99” showed almost no systemic colonization of apical shoot segments in an experiment with root-dip inoculation with *V. longisporum* isolate 43 (Figure 3C). The same was true for the late-flowering *A. thaliana* ecotype Bur (Figure 3D). The early flowering genotypes *B. alboglabra* “94” and *A. thaliana* ecotype L*er* were extensively colonized (Figure 3C,D). In *B. alboglabra*, resistance to systemic colonization was paralleled by a slower disease progression based on the symptoms of stunting and chlorosis (Figure 3E). In *A. thaliana*, stunting resistance as determined by the performance height [79] was increased in late-flowering ecotype Bur only during summer when light conditions in the greenhouse were optimal (Figure 3F). Several colonization resistance QTL were found in the vicinity of QTL controlling development time in *A. thaliana*, which explained the correlation of both traits [188]. A similar correlation was found in connection with the *V. longisporum* resistance QTL *Vet1* [189]. that also delayed development. To date it is not known whether both effects are controlled by the same or by closely linked gene(s). However, the correlation between longevity and resistance against some types of stress seems to be a general principle. Long-lived, late-flowering *A. thaliana* mutants have been shown to be more tolerant to oxidative stress caused by the herbicide paraquat, suggesting that longevity and oxidative stress tolerance are tightly linked [190]. Evidence for the role of oxidative stress in *V. longisporum* pathogenesis will be presented below (see Section 3.2.3.).

### 3.2. Pathogenesis Affects Host Development

#### 3.2.1. Pathogens Manipulate Host Development for Their Own Benefit

The most commonly described developmental implication of pathogenesis is the induction and exploitation of signaling pathways that benefit the pathogen, but are deleterious to the host [4,54,192]. Hormones or toxins produced by the pathogen may delay or promote senescence, depending on the lifestyle of the pathogen. The following examples illustrate how developmental pathways are exploited by pathogens and how plants can counter-steer during quantitative disease resistance.

Biotrophic and some hemibiotrophic pathogens preferentially produce or induce growth-promoting plant hormones, such as auxins or cytokinins, or change the host’s response to these hormones. The AvrRpt2 Type III effector of *P. syringae* DC3000 has been shown to increase auxin levels and auxin sensitivity in the host to promote disease in *A. thaliana* [193]. Upon infection by *Plasmodiophora brassicae*, a biotrophic protist causing clubroot disease in crucifers, cytokinins and auxins contribute to hypertrophy of root gall cells [194]. *P. brassicae* can produce cytokinins by itself [195] and induce transcriptomic changes in the host that lead to increased auxin and cytokinin contents in root galls [196]. Cytokinins also participate in the formation of green islands—green areas at the infection sites of obligate biotrophs like *P. graminis* on otherwise senescent host tissue [197]. While effectors of biotrophic pathogens promote disease in compatible interactions, host resistance in incompatible interactions is achieved by effector recognition and subsequent ETI based on HR cell death. ETI often results in complete resistance, which can, however, easily be overcome by mutations in the pathogen [198].

Necrotrophic and some hemibiotrophic pathogens promote senescence by targeting the respective signaling pathways. Two independent studies showed that some hemibiotrophic vascular pathogens can induce senescence-like processes by targeting the F-box protein COI1, which is part of the JA-binding JAZ-COI1-co-receptor [199,200]. *Fusarium oxysporum* as well as *V. longisporum* needed functional *Coi1* in the root to induce senescence symptoms during infection within the shoot of *A. thaliana*. *coi1* mutants showed a high level of quantitative resistance against both pathogens as inferred from disease symptoms, although host colonization still occurred. Strikingly, *coi1*-mediated resistance was independent of JA biosynthesis and JA-induced defense pathways as well as from SA-mediated defense [199,200]. Other pathogens targeting the JA-signaling pathway inhibit JA-mediated defenses via the JA-SA antagonistic crosstalk [201]. This seemed not to be the case in *coi1*-mediated resistance. COI1 was not a target of potential *V. longisporum*-produced JA-mimicks, since JA-mediated defenses were absent in *V. longisporum*-infected *delayed dehiscence 2* (*dde2*)-mutants that are impaired in JA biosynthesis [199]. Due to the suppression of pathogen-induced senescence, *coi1*-mutants were less colonized during the late stages of infection in both pathosystems [199,200].

NECROSIS AND ETHYLENE INDUCING PEPTIDE 1 (NEP1)-LIKE PROTEINs (NLPs) are widespread microbial toxins produced by necrotrophic and hemibiotrophic pathogens to induce cell death [202]. Hemibiotrophic *Moniliophthora perniciosa*, the causal pathogen of witches’ broom disease in cacao, initiated its necrotrophic phase and the production of NLP MpNEP2 upon carbon starvation in the apoplast [203]. In the preceding biotrophic phase, the pathogen increased sink strength in infected tissue possibly by producing cytokinins. This led to increased concentrations of extracellular hexoses in infected tissues and a quicker rise of intracellular sugars. Subsequent early senescence was accompanied by repression of photosynthesis. It was hypothesized that this strategy allows the pathogen to acquire soluble apoplastic carbohydrates during its biotrophic phase and to gain prime access to intracellular carbohydrates by killing senescent cells during its necrotrophic phase [203]. This example shows how the spatial and temporal control of signals can manipulate a host for the maximum benefit of the pathogen.

#### 3.2.2. “Developmental Buffering” Confers Quantitative Resistance to *V. longisporum* in *A. thaliana*

With very few exceptions, ETI is not an efficient resistance mechanism against necrotrophic or hemibiotrophic pathogens [4]. In contrast, toxins may elicit ETI and HR for the benefit of the necrotroph [204]—a process that has been termed effector-triggered susceptibility [205]. Resistance against hemibiotrophs and necrotrophs therefore relies on diverse other mechanisms that are often complex and not fully understood. In the following, the developmental effects of *V. longisporum* on its cruciferous hosts are described and signaling processes modulating susceptibility and quantitative resistance are presented.

**Figure 4:**
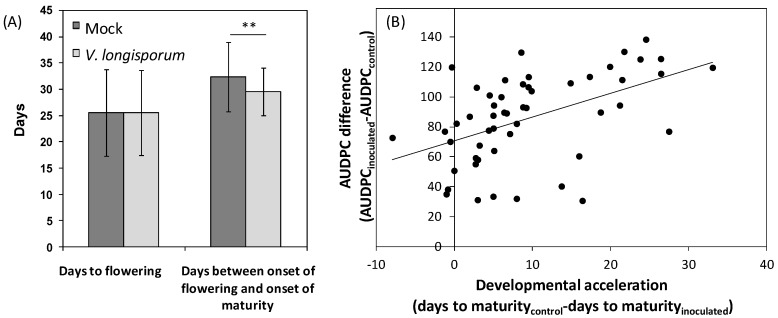
Developmental acceleration in *A. thaliana* and *B. alboglabra* after *V. longisporum* infection. **(A)** Flowering time and maturation time in a collection of 100 *A. thaliana* (Bur × L*er*)-recombinant inbred lines (RILs) after mock- and *V. longisporum* inoculation respectively. *N* = 94 to 102, each replicate corresponded to a batch of at least 15 plants. Asterisks denote a significant difference of *p* < 0.01 (*t*-test), error bars denote standard deviations. **(B)** Correlation between developmental acceleration by *V. longisporum* and disease progression, expressed as AUDPC difference between inoculated and mock-inoculated plants in *B. alboglabra* (94 × 99)-F3-families. *N* = 52, *r* = 0.49, *p* < 0.01. Each data point represents one F3-family, differences were calculated from means of 38 inoculated and 11 mock-inoculated plants in each F3-family.

*V. longisporum* induced premature senescence in *A. thaliana* and *B. alboglabra* in greenhouse experiments (Figure 4). This finding is in accordance with observations on oilseed rape in the field [206]. Premature senescence in *A. thaliana* was not due to an acceleration of flowering time, but reduced the time from flowering to maturity (Figure 4A). Acceleration of maturity was observed in a population of (Bur × L*er*)-recombinant inbred lines (RILs). QTL controlling resistance against premature senescence, however, could not be identified. In *B. alboglabra* we showed that acceleration of maturity was positively correlated with disease progression (Figure 4B). These results suggest, in accordance with theories for necrotrophic pathogens, that acceleration of maturity was mainly exerted during the necrotrophic, systemic phase of infection and that premature senescence is a disease symptom and not a result of defense signaling.

**Figure 5:**
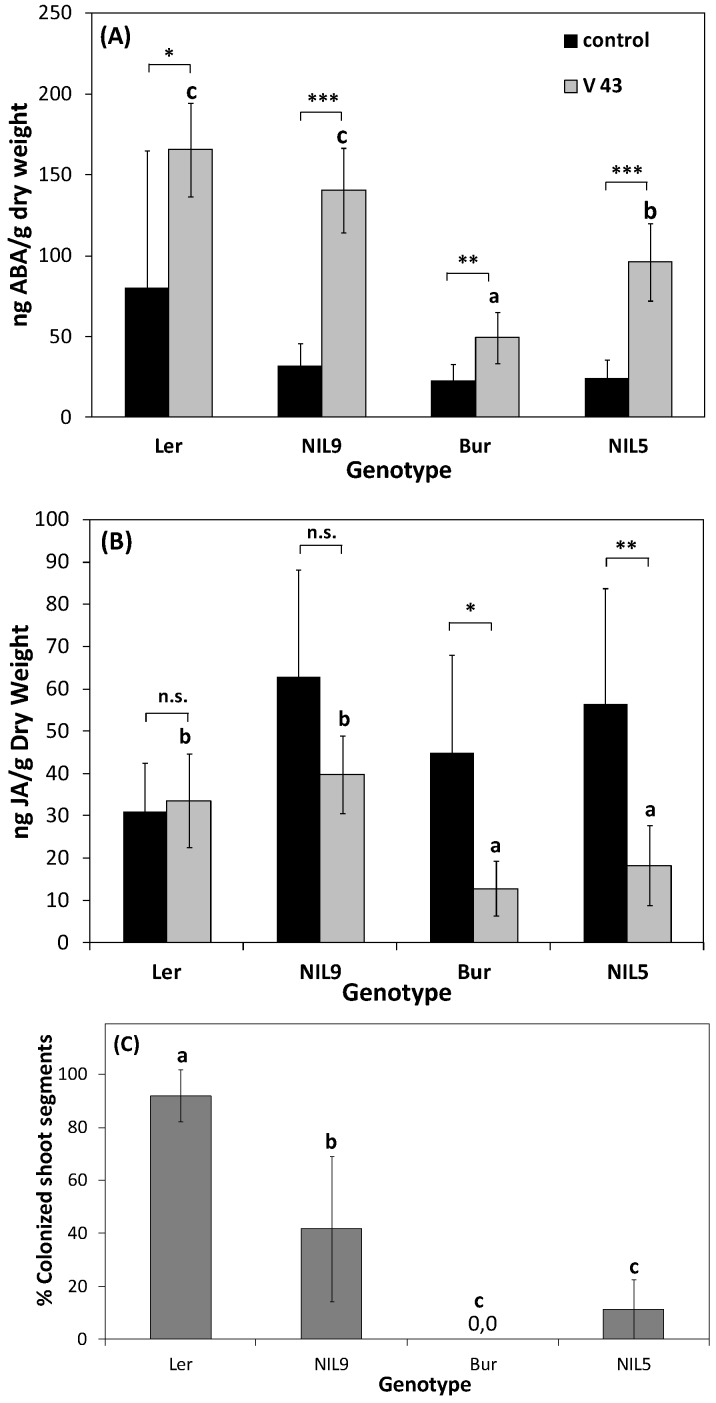
Hormone contents and systemic colonization by *V. longisporum* in *A. thaliana* ecotypes L*er*, Bur, and near-isogenic lines differing in a region containing the colonization resistance QTL *vec1* on chromosome 2. Contents of (**A**) abscisic acid (ABA) and (**B**) jasmonic acid (JA) in Bur, L*er* and two (Bur × L*er*)-near-isogenic lines (NILs): NIL9 (L*er* alleles in the variable region) and NIL5 (Bur alleles). Asterisks refer to the significance level of differences between mock-inoculated and *V. longisporum*-treated plants within one genotype (*t*-test). Mean contents of inoculated plants marked with different letters differed significantly at *p* < 0.05. Mean phytohormone contents of controls did not differ significantly (one-way ANOVA and post-hoc Tukey test). *N* = 6–7, each replicate corresponded to a batch of 15 plants. Vertical bars denote standard deviations (data from [79]). (C) Systemic colonization by *V. longisporum* in the same plants that have been used for phytohormone measurements. Means marked with different letters differed at *p* < 0.05 (one-way ANOVA and post-hoc Tukey test). *N* = 6–7, each replicate corresponded to a batch of 15 plants. Vertical bars denote standard deviations.

Comparing a resistant and a susceptible genotype with respect to individual signaling pathways can give information about components involved in resistance and susceptibility as well as in developmental processes. For this purpose, phytohormone analyses and gene expression studies were performed in near-isogenic lines originating from the *A. thaliana* ecotypes Bur and L*er*. The lines were polymorphic for a genomic region harboring the major resistance QTL *vec1* and differed in resistance to systemic colonization by *V. longisporum.* Previous studies showed a disease-promoting effect for ABA and JA at the stage of silique maturity as inferred from phytohormone contents of Bur, L*er*, and two NILs representing the respective alleles in *vec1* (Figure 5, [79]). NIL5, carrying alleles of the resistant parent Bur in a ~3 Megabase region of *vec1*, showed less severe ABA-induction and suppression of JA-production after *V. longisporum* infection in shoots of maturing plants [79].

#### 3.2.3. Transcriptional Response to *V. longisporum* at Maturity in a Susceptible and a Resistant *A. thaliana* Line

**Figure 6:**
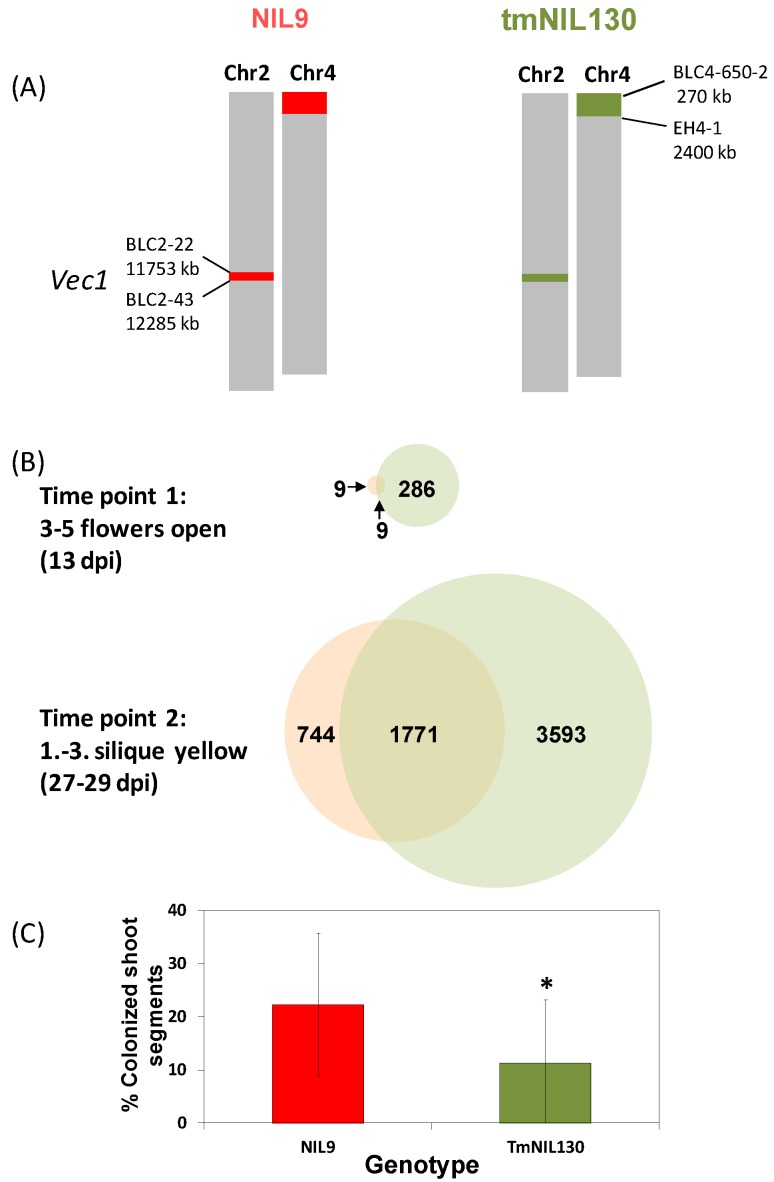
Differential gene expression and systemic colonization after *V. longisporum* infection in two (Bur × L*er*)-NILs differing in a region within the colonization resistance QTL *vec1*. (**A**) Genotype of NIL9 and tmNIL130 in the variable regions. Red parts stand for L*er* alleles, green parts for Bur alleles in the variable regions. Grey parts are isogenic with respect to the tested marker loci (for marker grid in isogenic regions see [79]). Names and physical positions in kilobases (kb) of markers delimiting variable regions are given next to the bars representing chromosomes. For primer information see Supplementary File Table S1; (**B**) Genes differentially expressed by *V. longisporum* infection in the hypocotyl-shoot-transition zone of NIL9 and tmNIL130 at two time points after infection. For growth-, inoculation- and RNA extraction protocol see GEO accession GSE70021. Hybridization to Affymetrix AraGene-1_1-st was performed by the Nottingham Arabidopsis Stock Centre (NASC) Affymetrix service [212]. Data were rma- (robust multichip average) normalized using the Bioconductor package affy [213], and differential expression was calculated using linear models with the Bioconductor package limma [214] by pairwise comparisons. Annotations were provided by the Bioconductor pd.aragene.1.1.st annotation package [215]. Only expression differences with a significance level of *p* < 0.05 were regarded at the probeset level. If more than one probeset represented a transcript cluster (≙ mRNA), transcript cluster means were calculated from values for individual probesets; (**C**) Systemic colonization of NIL9 and tmNIL130 at time point 2 (onset of silique maturity). *N* = 12, *t*-test. Samples were taken from 30 plants per replicate, among which were also the plants sampled for microarray analysis.

A microarray analysis was performed to monitor genome-wide transcriptomic changes in response to *V. longisporum* controlled by *vec1*. For this purpose, a tailor-made near-isogenic line (tmNIL130) has been selected that contained a max. 532 kb- introgression of Bur-alleles surrounded by L*er*-alleles in *vec1* (Figure 6A). The introgression was designed to be as small as possible while still controlling a significant level of resistance (Figure 6C). The transcriptional response to *V. longisporum* in tmNIL130 was compared to the one in NIL9 that carried L*er*-alleles in the indicated region. In all other regions, the two lines were isogenic at the genotyped marker loci except for a region at the top of chromosome 4 (Figure 6A). Differential gene expression was studied in samples containing the hypocotyl and the shoot basis, since colonization resistance has been reported to be effective in the hypocotyl of *Brassica* spp. [207]. Thus, local and systemic responses were to be determined. Samples were collected during the two crucial stages of *V. longisporum* colonization (Figure 6B): shortly after the onset of flowering when the shoot started elongation and systemic colonization was shown to start (time point 1, 13 days post inoculation, dpi), and upon maturation of the first silique when extensive proliferation was shown to occur in susceptible genotypes (time point 2, 27–29 dpi). The complete data set and the metadata containing all experimental descriptions have been released to Gene Expression Omnibus (GEO, [208]) repository at the National Center for Biotechnology Information (NCBI) under the reference number GSE70021 [209]. 

Generally, partially resistant tmNIL130 was characterized by a much more pronounced transcriptional response to *V. longisporum* infection compared to NIL9 (Figure 6B). At the onset of flowering, only 18 transcripts were differentially expressed after *V. longisporum* challenge in NIL9, but 295 transcripts were mostly up-regulated by *V. longisporum* in tmNIL130. Among them were many defense-related genes like the transcription factor WRKY33, which was shown to mediate JA/ET-dependent defense responses against necrotrophs [210] with 2.6-fold up-regulation, or *Frk1*, a PTI marker gene [211] with 8.6-fold up-regulation after infection. At the onset of maturity, massive transcriptional reprogramming has occurred in both lines, but in tmNIL130 still more than twice as many genes were differentially expressed compared to NIL9.

To identify processes involved in defense and/or senescence, MAPMAN [216] was applied to sort the genes that were differentially expressed at the onset of maturity on the basis of their annotation (Table 1 and Table 2). Up- and down-regulated genes in both lines with roles in phytohormone signaling, the response to oxidative stress as well as WRKY transcription factors were compiled. Four major trends were identified:

**(1) Ethylene-associated transcripts showed a shift towards defense-relevant branches in tmNIL130.**

Genes involved in ET synthesis, signaling and response were predominantly up-regulated after *V. longisporum* infection in both lines. Expression changes were more pronounced in tmNIL130. ACC-synthases (ACS) were more strongly up-regulated in tmNIL130 and interestingly, the catalytically inactive isoform ACS1 was only up-regulated in tmNIL130. ACS1-expression was previously associated with young tissues only [217]. ET receptors were induced only in tmNIL130. It must be kept in mind that owing to the inhibitory nature of the ET receptors, increasing their density decreases sensitivity to the hormone. Transcription factor ERF14, which acts in the ERF1/ORA59 defense signaling branch of JA and ET [218], was strongly up-regulated in tmNIL130. ORA59, however, was down-regulated in NIL9, not in tmNIL130 (Table 1). These findings suggest that the defense branch of ET signaling was strengthened after infection in tmNIL130, while it was weakened in NIL9.

**(2) Many genes acting in auxin synthesis, homeostasis, transport and response were differentially expressed upon infection in tmNIL130.**

From the sheer number of down-regulated genes in tmNIL130 it could be concluded that auxin played a negative role in *V. longisporum* resistance (Table 1). However, the auxin biosynthesis gene NIT2 was 16-fold up-regulated in tmNIL130. Auxin-conjugating enzymes were also up-regulated to a considerable degree, among them GH3.2, which has been shown to mediate susceptibility to *B. cinerea* and *P. syringae* [123]. However, with tmNIL130 being the resistant line, this mechanism did not seem to apply to *V. longisporum* infection. Auxin induction and tight control at the same time point to a sophisticated and specialized role of auxin signaling in mediating *V. longisporum* resistance during the late stage of infection at the onset of maturity.

**(3) Several defense-related WRKY transcription factors were exclusively up-regulated in tmNIL130.**

Several WRKY transcription factors with sometimes seemingly contrasting roles were exclusively up-regulated in tmNIL130 by *V. longisporum* infection (Table 1). Among the most strongly up-regulated WRKY transcription factors were WRKY40 and WRKY60 as positive regulators of defense against necrotrophs and negative regulators of ABA signaling [140]. However, the 6-fold up-regulated WRKY63 has been reported to positively regulate some ABA-responses [219]. Highly induced WRKY62 as well as WRKY70 and WRKY53 have been shown to positively regulate basal defense [136,139]. WRKY53 and WRKY6 are also positive regulators of senescence. Interestingly, moderately up-regulated WRKY57 was shown to mediate JA-auxin cross-talk, inhibiting JA-induced senescence and promoting auxin-signaling [106]. This would be in accordance with the finding that JA played a disease-promoting role during the late stage of infection as concluded from phytohormone analyses (Figure 5). Considering their emerging role as molecular switches between pathways, WRKY transcription factors possibly contributed to a much more elaborate control of the defense—senescence signaling network in tmNIL130 at the onset of maturity compared to NIL9.

**Table 1:** Regulatory pathway genes differentially expressed by *V. longisporum* infection in the hypocotyl and basal shoot of two *A. thaliana* (Bur × L*er*) near-isogenic lines (NILs) at the onset of silique maturity in a microarray experiment.

NIL9		tmNIL130			
Gene	FC	Gene	FC	Gene	FC
**Ethylene biosynthesis, -signaling and -response**					
at2g25450	6.94	at5g20400	2.13	at5g44210 (ERF9)	2.59
at4g37770 (ACS8)	4.07	at5g59540	2.48	at3g23150 (ETR2)	2.46
at5g65800 (ACS5)	3.90	at5g43440	2.18	at1g04310 (ERS2)	2.84
at1g01480(ACS2)	3.59	at1g03400	2.10	at3g50260 (CEJ1)	2.84
at4g26200 (ACS7)	3.18	at5g43450	2.13	at2g40940 (ERS1)	2.40
at5g44210 (ERF9)	2.94	at2g25450	12.06	at5g25190	3.67
at5g25190	5.09	at4g37770 (ACS8)	4.99	at3g11930	2.25
at4g37580 (HLS1)	2.07	at5g65800(ACS5)	3.88	at1g04370 (ATERF14)	4.98
at3g11930	3.67	at1g01480 (ACS2)	11.90	at1g55150	3.49
at1g55150	2.10	at3g61510 (ACS1)	5.00	at1g09740	2.32
at3g20640	2.40	at4g26200 (ACS7)	2.17		
at1g77330	0.28	at2g31230 (ATERF15)	0.40		
at5g07580	0.38	at2g20100	0.45		
at2g31230 (ATERF15)	0.42	at1g27660	0.32		
at1g06160 (ORA59)	0.33	at1g49830	0.33		
at5g44350	0.21	at4g29100	0.48		
at1g27660	0.37				
**Jasmonic acid biosynthesis, signaling and response**					
		at1g18020	2.89		
		at1g52070	2.64		
		at5g48180 (NSP5)	2.25		
at1g55020 (LOX1)	0.44	at1g09400	0.35		
at1g76690 (OPR2)	0.39	at3g16470 (JR1)	0.24		
at3g16470 (JR1)	0.34	at3g16450	0.40		
at3g16450	0.35				
**Salicylic acid biosynthesis, signaling and response**					
at1g66720	6.23	at1g66720	12.03		
at5g04370 (NAMT1)	0.18	at5g38020	0.20	at4g26420 (GAMT1)	0.35
at5g37990	0.09	at1g05670	0.38	at5g37990	0.08
		at5g04370 (NAMT1)	0.45		
**Abscisic acid biosynthesis, signaling and response**					
at4g18350 (NCED2)	4.26	at4g18350 (NCED2)	10.1	at5g23350	2.39
at5g50720 (ATHVA22E)	2.03	at1g30100 (NCED5)	13.32	at5g50720 (ATHVA22E)	2.31
at1g74520 (ATHVA22A)	2.84	at3g24650 (ABI3)	8.23		
at4g32810 (CCD8)	0.41	at2g36020 (HVA22J)	0.20	at2g17770 (ATBZIP27)	0.28
at2g44990 (CCD7)	0.15	at4g32810 (CCD8)	0.28	at5g62490 (ATHVA22B)	0.49
at3g63210 (MARD1)	0.50	at2g44990 (CCD7)	0.16	at1g45249 (ABF2)	0.39
at2g17770 (ATBZIP27)	0.32	at3g43600 (AAO2)	0.26		
at5g23370	0.46	at1g52920 (GCR2)	0.48		
at1g45249 (ABF2)	0.36	at3g63210 (MARD1)	0.45		
**Brassinosteroid synthesis, signaling and response**					
at3g50660 (DWF4)	4.09	at3g50660 (DWF4)	2.99	at2g22830 (SQE2)	2.28
		at1g76090 (SMT3)	2.30	at4g36780	2.16
		at4g37760 (SQE3)	0.34		
**Cytokinin biosynthesis, signaling and response**					
at5g05860 (UGT76C2)	2.34	at5g05860 (UGT76C2)	2.06	at1g22400 (UGT85A1)	2.72
at2g17820 (AHK1)	2.10	at4g29740 (CKX4)	5.18		
at2g47430 (CKI1)	0.34	at5g35750 (AHK2)	0.45	at2g01830 (AHK4)	0.44
at2g01830 (AHK4)	0.44	at2g25180 (ARR12)	0.32		
**Gibberellic acid biosynthesis, signaling and response**					
at5g59845	6.82	at1g02400 (ATGA2OX4)	3.05		
		at1g75750 (GASA1)	10.66		
		at5g59845	5.97		
at1g52820	0.24	at4g23340	0.33	at5g51810 (GA20OX2)	0.22
at4g25420 GA5	0.22	at4g25420 (GA5)	0.33	at1g14920 (GAI)	0.34
at1g14920 (GAI)	0.42				
**Auxin biosynthesis, signaling and response**					
at5g13360	2.46	at3g44300 (NIT2)	16.35	at5g54510 (GH3.6)	3.00
at1g10810	4.09	at5g55250 (IAMT1)	2.62	at4g36110	4.11
at5g13320 (PBS3)	4.09	at3g15450	6.61	at1g17345	2.57
at5g54510 GH3.6	2.78	at2g23170 (GH3.3)	15.67	at4g37390 (YDK1/GH3.2)	5.77
at1g17345	2.48	at1g10810	3.26	at2g04850	2.06
at2g04850	2.69	at5g13320 (PBS3)	6.08	at4g12410	2.09
at4g12410	3.37	at1g60730	2.48	at5g13370	4.57
		at5g01100	2.93	at4g27450	3.94
at1g77690 (LAX3)	0.37	at3g10870 (MES17)	0.49	at3g12955	0.34
at5g47530	0.29	at1g05560 (UGT1)	0.45	at3g02250	0.45
at4g17280	0.17	at1g77690 (LAX3)	0.42	at4g00880	0.30
at1g16510	0.48	at5g16530 (PIN5)	0.42	at1g19830	0.26
at2g14960	0.24	at1g73590 (PIN1)	0.47	at3g61900	0.45
at3g02250	0.46	at5g47530	0.21	at1g14970	0.31
at4g00880	0.46	at4g17280	0.21	at3g61750	0.25
at3g61750	0.34	at3g43120	0.34	at5g65470	0.41
at1g28130 (GH3.17)	0.27	at3g47620 (AtTCP14)	0.40	at4g34750 (SAUR_E)	0.31
		at3g25290	0.31	at2g04852	0.49
		at2g34680 (AIR9)	0.42	at4g12980	0.39
**WRKY transcription factors **					
at4g31800 (WRKY18)	2.38	at1g69810 (WRKY36)	2.05	at1g69310 (WRKY57)	1.69
at5g46350 (WRKY8)	3.85	at4g31800 (WRKY18)	2.76	at3g56400 (WRKY70)	2.48
at2g25000 (WRKY60)	4.09	at5g46350 (WRKY8)	4.19	at5g64810 (WRKY51)	4.65
at1g80590 (WRKY66)	4.13	at1g66600 (WRKY63)	6.33	at1g80590 (WRKY66)	3.18
at2g47260 (WRKY23)	4.09	at4g23810 (WRKY53)	3.01	at5g26170 (ATWRKY50)	2.96
at4g22070 (WRKY31)	3.19	at2g23320 (WRKY15)	2.31	at5g49520 (WRKY48)	1.90
		at4g23550 (WRKY29)	2.83	at2g47260 (WRKY23)	6.37
		at2g25000 (WRKY60)	3.91	at4g22070 (WRKY31)	4.67
		at1g18860 (WRKY61)	4.38	at1g62300 (WRKY6)	3.73
		at5g13080 (WRKY75)	3.47	at5g24110 (WRKY30)	3.15
		at5g01900 (WRKY62)	5.49	at1g80840 (WRKY40)	6.85
at5g52830 (WRKY27)	0.45	at4g39410 (WRKY13)	0.50	at2g37260 (WRKY44)	0.34
at3g58720	0.48	at5g52830 (WRKY27)	0.34	at2g44745	0.47
at2g34830 (WRKY35)	0.46	at2g34830 (WRKY35)	0.44		

Classification of transcripts with MAPMAN [216]. In each class, differentially expressed genes in the susceptible NIL9 are displayed on the left side, those in resistant tmNIL130 on the right side. Up-regulated genes are at the top of each group, down-regulated genes at the bottom. Only those transcripts were included whose fold change (FC) was over 2 or below 0.5. Means for transcripts (represented by transcript clusters in AraGene-1_1-st microarrays) were calculated from individual probeset values, for which the cutoff was set at *p* < 0.05.

**Table 2:** Genes involved in the response to oxidative stress differentially expressed by *V. longisporum* infection in the hypocotyl and basal shoot of two *A. thaliana* (Bur × L*er*) near-isogenic lines (NILs) at the onset of silique maturity in a microarray experiment.

NIL9		tmNIL130			
Gene	FC	Gene	FC	Gene	FC
**Redox homeostasis**					
at1g69880 (ATH8)	5.95	at1g69880 (ATH8)	7.52	at3g02870 (VTC4)	2.60
at5g61440 (ACHT5)	2.79	at1g19730 (ATH4)	3.45	at1g63460	2.04
at4g39830	4.77	at5g61440 (ACHT5)	3.45	at2g31570 (ATGPX2)	2.45
at1g20630	2.82	at5g16400 (TRXF2)	2.46	at2g16060 (GLB1)	3.65
		at1g21750 (ATPDIL1-1)	2.58	at1g03850	5.63
		at3g19010	2.40	at4g15700	4.01
		at2g43350 (ATGPX3)	2.24	at1g32760	2.01
		at4g39830	5.01	at1g20630 (CAT1)	2.62
		at4g09010 (APX4)	3.47	at5g17770 (CBR1)	2.30
		at4g08390 (SAPX)	2.54		
at4g33040	0.44	at3g20560 (ATPDIL5-3)	0.48	at4g33040	0.31
at5g11930	0.33	at4g18260	0.16	at5g11930	0.26
		at3g19000	0.44	at2g47870	0.41
		at5g21100	0.37	at4g25100 (FSD1)	0.23
		at2g47880	0.24	at1g31170 (SRX)	0.45
**Peroxidases**					
at3g49120 (ATPERX34)	2.51	at5g19880	2.53	at1g49570	5.56
at2g37130 (PER21)	4.44	at5g19890	4.38	at2g34060	2.20
at1g71695 (PER12)	2.29	at3g49120 (ATPERX34)	2.36	at5g64110	4.40
at5g15180	6.40	at2g37130 (PER21)	3.78	at5g64100	6.12
at5g64110	3.77	at1g71695 (PER12)	3.00	at5g05340	3.91
at5g51890	24.95	at4g37520 (PER50)	2.98	at4g33420	3.14
		at5g64120	11.16	at5g51890	18.62
		at5g39580	6.27	at1g05240	2.48
		at5g15180	4.10		
at5g67400	0.09	at1g68850	0.26		
at5g14130	0.31	at5g42180 (PER64)	0.21		
at5g06720	0.46				
at1g30870	0.33				
at5g42180 (PER64)	0.26				
at1g44970	0.11				
at1g05250	0.15				
at1g05240	0.13				
**Glutathione-S-transferases**					
at5g02780	7.93	at1g02920 (ATGSTF7)	4.38	at1g14550	5.27
at2g29440 (ATGSTU6)	6.41	at2g29470 (ATGSTU3)	8.22	at1g14540	6.54
at3g09270: (ATGSTU8)	3.21	at4g19880	2.23	at3g09270 (ATGSTU8)	3.94
at1g17180 (ATGSTU25)	3.02	at1g17170 (ATGSTU24)	2.26	at1g17180 (ATGSTU25)	2.19
at1g69930 (ATGSTU11)	2.05	at5g02780	3.33	at1g69930: (ATGSTU11)	6.67
		at2g29440 (ATGSTU6)	8.74	at1g02930 (ATGSTF6)	5.20
		at4g02520 (ATGSTF2)	2.74	at2g29480 (ATGSTU2)	4.03
		at2g02930 (ATGSTF3)	4.33	at2g29450 (ATGSTU5)	2.83
		at1g69920 (ATGSTU12)	5.13		
		at1g74590 (ATGSTU10)	4.16		
at1g49860 (ATGSTF14)	0.19	at3g03190 (ATGSTF11)	0.27		
at1g78370 (ATGSTU20)	0.20	at1g78370 (ATGSTU20)	0.24		
at1g10360 (ATGSTU18)	0.44				
at5g62480: (ATGSTU9)	0.39				
at5g17220: (ATGSTF12)	0.10				

Classification of transcripts with MAPMAN [216]. In each class, differentially expressed genes in the susceptible NIL9 are displayed on the left side, those in resistant tmNIL130 on the right side. Up-regulated genes are at the top of each group, down-regulated genes at the bottom. Only those transcripts were included whose fold change (FC) was over 2 or below 0.5. Means for transcripts (represented by transcript clusters in AraGene-1_1-st microarrays) were calculated from individual probeset values, for which the cutoff was set at *p* < 0.05.

**(4) tmNIL130 showed a much stronger response to oxidative stress.**

The differential expression of genes involved in oxidative stress response was perhaps the most conspicuous difference between NIL9 and tmNIL130 (Table 2). While in NIL9, only few ROS-detoxifying enzymes responded to infection, most of which were down-regulated, tmNIL130 showed up-regulation of a large portfolio of enzymes involved in redox homeostasis and ROS metabolism like catalase and peroxidases. A much greater diversity of glutathione-S-transferases, which are generally thought to detoxify oxidation products in the cell [220], were up-regulated in tmNIL130. Keeping in mind that these processes took place during silique maturity and the late phase of *V. longisporum* infection, this reaction clearly points to a stringent control of cell death and senescence in tmNIL130, obviously restricting pathogen growth.

## 4. Concluding Remarks

**Figure 7:**
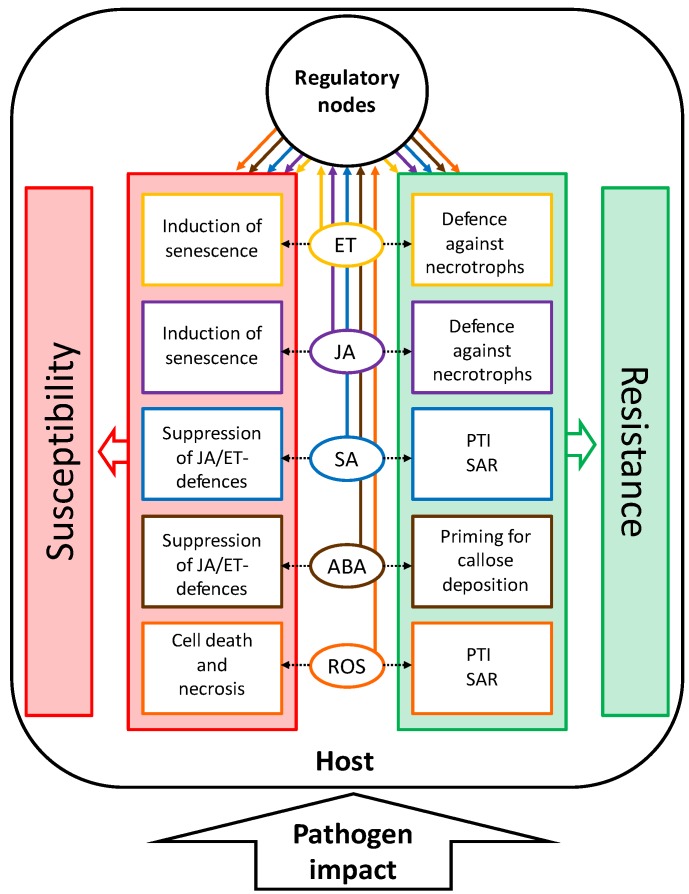
A model for the roles of potentially senescence-inducing signaling molecules during necrotrophic host-pathogen-interactions. Ethylene (ET), jasmonic acid (JA), salicylic acid (SA), abscisic acid (ABA) and reactive oxygen species (ROS) are usually induced during necrotrophic interactions. If the senescence-promoting effects prevail, host susceptibility will be increased. If the host succeeds to restrict signaling events to defence-related branches, resistance can be achieved. Regulatory nodes, such as transcription factors, may act as molecular switches between signaling branches. Colored arrows symbolize signaling pathways triggered by different signaling molecules. Dashed arrows symbolize potential responses. PTI = PAMP-triggered immunity, SAR = systemic acquired resistance, PAMP = pathogen-associated molecular pattern.

Plant senescence and host response to pathogen infection are both associated with numerous signaling events and major transcriptional changes. Considering the fact that signaling pathways overlap and often the same molecules are involved in both senescence and response to biotic stress, one might be inclined to conclude that some developmental changes inevitably occur upon pathogen infection. Recent work has greatly increased our knowledge of signal integration, crosstalk between signaling pathways and the fine-tuning of stress responses and developmental processes. The emerging picture suggests that in most cases distinct branches of a signaling pathway control different developmental and defense responses. Some signaling components act as regulatory nodes to channel the plant’s response into a certain direction. The capacity of the plant to control such responses may decide over disease or resistance especially in quantitative disease resistance, which is the more common type of resistance to necrotrophic and hemibiotrophic pathogens. The interaction of necrotrophic and hemibiotrophic pathogens with their hosts is characterized by extensive transcriptional changes and the response of the majority of signaling pathways available to plants. Often these changes lead to the induction of senescence and cell death, which is favorable to a necrotrophic lifestyle based on the killing of cells. While pathogen-induced senescence relies on the induction of the “wrong” signaling components, the capacity of the plant to buffer these interferences and channel the signaling pathways into the “right” responses seems to account for a large part of quantitative and developmentally influenced resistance. This capacity may be pre-formed: A certain developmental type or stage is *per se* less susceptible to pathogen manipulations because the targets are not available or the cellular environment is otherwise hostile to the pathogen. During infection, much depends on induced responses in the host: Resistance can be achieved by counteracting senescence and cell death and by activating defense responses (Figure 7).

Multiple mechanisms may underlie an adequate control of signaling events: The timing and intensity of the response, the concentration of signaling molecules or the tissue-specific expression of genes for hormone synthesis, receptors, transporters or responsive genes. It is a challenge for the future to identify regulators that control developmental homeostasis and enable the plant to stay on the narrow path of an adequate response to pathogens.

## Supplementary Files

Supplementary File 1
