# Supplementary material for: Keeping Control: The Role of Senescence and Development in Plant Pathogenesis and Defense
Source: Plants (Basel). 2015 Jul 13;4(3):449–88. doi: 10.3390/plants4030449 (PMC4844401; doi:10.3390/plants4030449)
Supplement: Supplementary File 1 [file plants-04-00449-s001.pdf]

Supplementary File

Table S1. Marker information for genotyping (Bur × L*er*) near-isogenic lines NIL9 and tmNIL130 at variable genomic regions.

Marker Name	Chr.	Position (kb)AGI	Forward Primer	T_m (℃)	Reverse Primer	T_m (℃)	T_a (℃)	t_e (s)	Restr. Enzyme	Allelic state NIL9	Allelic state tmNIL130
BLC2-22	2	11753	GAGATATGTGGTTCAAGTCT	56	AACTCTACGACGCAGATCC	58	55	40	MnlI	Ler	Ler
BLC2-25	2	11799	GGAGGGTCCACCACTCGGC	66	GGCGGAAGGAAGAAAAACACG	64	55	40	HinfI	Ler	Bur
BLC2-42	2	12270	CCGATGAAGAAGAGAGATTG	58	CACAACGGTCGGATCACGGAT	66	55	40	NlaIV	Ler	Bur
BLC2-43	2	12285	CTTTCCCTTCGTGATTCGTTAC	64	GCTAATCCTCGTCTTGCTCTGT	66	55	40	DdeI	Ler	Ler
BLC4-650-2	4	270	CAAAGGTGGTTCCTTTTGTTG	60	TGCATTCCACGCTTGATACT	58	55	40	NdeI	Ler	Bur
EH4-1	4	2400	See Reference [188]							Bur	Bur

Abbreviations: Chr. = chromosome, kb = kilobases, AGI = Arabidopsis Genome Initiative, T_m = melting temperature, T_a = annealing temperature, t_e = elongation time, restr. = restriction.
